# DNA-Loaded Cationic Liposomes Efficiently Function as a Vaccine against Malarial Proteins

**DOI:** 10.1016/j.omtm.2017.08.004

**Published:** 2017-08-23

**Authors:** Wesley L. Fotoran, Rachele Santangelo, Beatriz N.M. de Miranda, Darrell J. Irvine, Gerhard Wunderlich

**Affiliations:** 1 Department of Parasitology, Institute for Biomedical Sciences, University of São Paulo, Av. Prof. Lineu Prestes 1374, São Paulo, 05508000, Brazil; 2 Institute of Chemistry of São Carlos, University of São Paulo, Av. Trabalhador São-Carlense 400, São Carlos, 13566-590, Brazil; 3 Koch Institute for Integrative Cancer Research and Department of Biomedical Engineering and Department of Materials Science and Engineering, Massachusetts Institute of Technology, Cambridge, MA, USA; 4 Ragon Institute of MGH, MIT and Harvard University, Boston, MA, USA; 5 Howard Hughes Medical Institute, Chevy Chase, MD, USA

**Keywords:** cationic liposomes, DNA vaccine, malaria

## Abstract

The delivery of antigens as DNA vaccines is an efficient alternative to induce immune responses against antigens, which are difficult to produce in recombinant form. However, the delivery of naked DNA is ineffective or relies on sophisticated ballistic devices. Here, we show a combination of liposome application and naked DNA vaccine that successfully overcomes these problems. Upon entrapment of plasmids encoding different antigens in cationic particles, transfection efficiencies similar to commercial kits were achieved in in vitro cell cultures. The liposome-based approach provided strong humoral responses against three malarial antigens, namely the Circumsporozoite protein and the C terminus of merozoite surface protein 1 from *Plasmodium vivax* (titers 10^4^ or 10^3^–10^4^, respectively) and *P. falciparum* Rhoptry antigen 5 from *Plasmodium falciparum* (titers 10^3^–10^4^). When employed in *P. falciparum* growth-inhibition assays, antibodies demonstrated consistent reinvasion-blocking activities that were dose dependent. Liposome-formulated DNA vaccines may prove useful when targets cannot be produced as recombinant proteins and when conformation-dependent and highly specific antibodies are mandatory.

## Graphical Abstract

**Figure:**
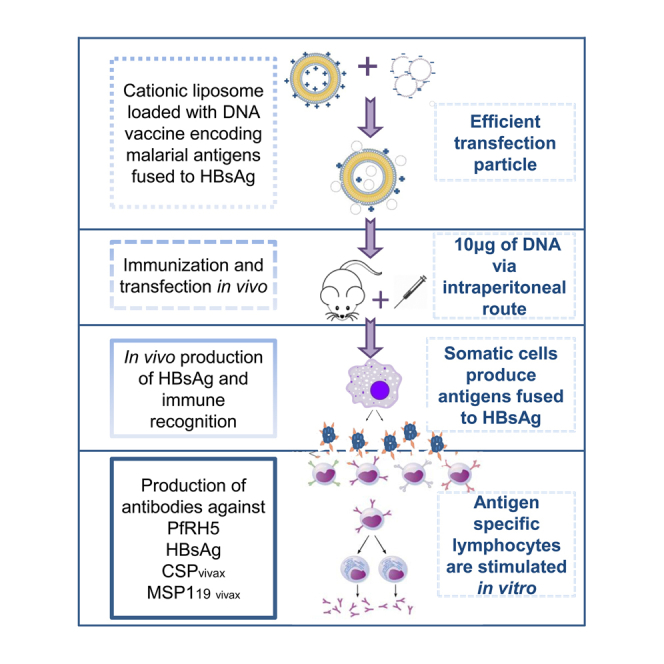

## Introduction

Gene therapy has developed into a valuable tool for many applications.^1^ One of these is the use of genes in DNA vaccines,^2^ and the perhaps most important advantage is the facilitated production of DNA in good manufacturing practice quality instead of the often cumbersome production of correctly folded recombinant antigen or its production in modified virus.^3^ However, DNA vaccines and recombinant virus have different drawbacks for application as vaccines. For example, the amount of DNA to be used in naked DNA vaccines normally exceeds the quantity of recombinant protein that would be used for the same purpose. For example, while the Alum-adjuvanted hepatitis B virus (HBV) vaccine contains 20 μg protein per dose for adults, the same antigen delivered as experimental DNA vaccine was injected in 400 μg doses in *Aotus* monkeys, and its immunogenicity was much lower than that of recombinant protein.^4^ Also, the number of efficiently loaded cells that then initiate the production of the vaccine antigen is considerably low,^2^ leading to a modest immune response. Recombinant viruses, although highly effective and immunogenic, cause an immune response against themselves that then hampers effective boosting against the vaccine antigen.^5^ The latter effect can be partially overcome with prime-boost protocols using different virus platforms or prime-boost protocols using recombinant virus priming followed by a recombinant protein or recombinant virus boost (see, for example, Dale et al.^6^). Both issues are significant points to consider when planning vaccines.^5^, ^6^

Another relevant aspect is the design of a DNA vaccine vector to enhance immunological responses.^7^ Targets without strong T helper cell epitopes may be tagged to universal T cell epitopes such as PADRE.^8^ A further approach is the fusion of the target antigen to a particle-forming peptide that then presents the target on virus-like particles or virosomes (reviewed in Grgacic and Anderson^9^). In this context, we designed a model based on the malaria vaccine that is most advanced in its development, RTS/S.^10^ We fused several vaccine-relevant antigen-encoding genes to the gene encoding the small hepatitis B surface protein, which itself forms empty virosomes. We included classical vaccine-candidate antigens, such as the repeat region of the circumsporozoite protein of *Plasmodium vivax* (PvCS), the C terminus of merozoite surface protein 1 (MSP1_19_) of *P. vivax*, as well as the hard-to-express *Plasmodium falciparum* Rhoptry protein 5 (PfRH5), the probably most promising candidate vaccine target against *P. falciparum* blood stages to date.^11^ To enhance the potential of DNA vaccination without the use of sophisticated technology, such as ballistic devices or microneedles, we loaded the recombinant plasmid DNAs in fusogenic liposomes. The total amount of vaccine DNA used here is about 10 times less than what is used in conventional, needle intramuscular-delivered naked DNA vaccines.^12^ As a proof of principle, we use the antisera against the PfRH5 antigen produced in mice to obtain strong *Plasmodium* reinvasion inhibition, comparable to what was achieved with adenoviral vectors.

## Results

### Cationic Liposomes Entrap Plasmids and Transfect In Vitro at an Efficiency Similar to Commercial Transfection Reagents

**Figure 1:**
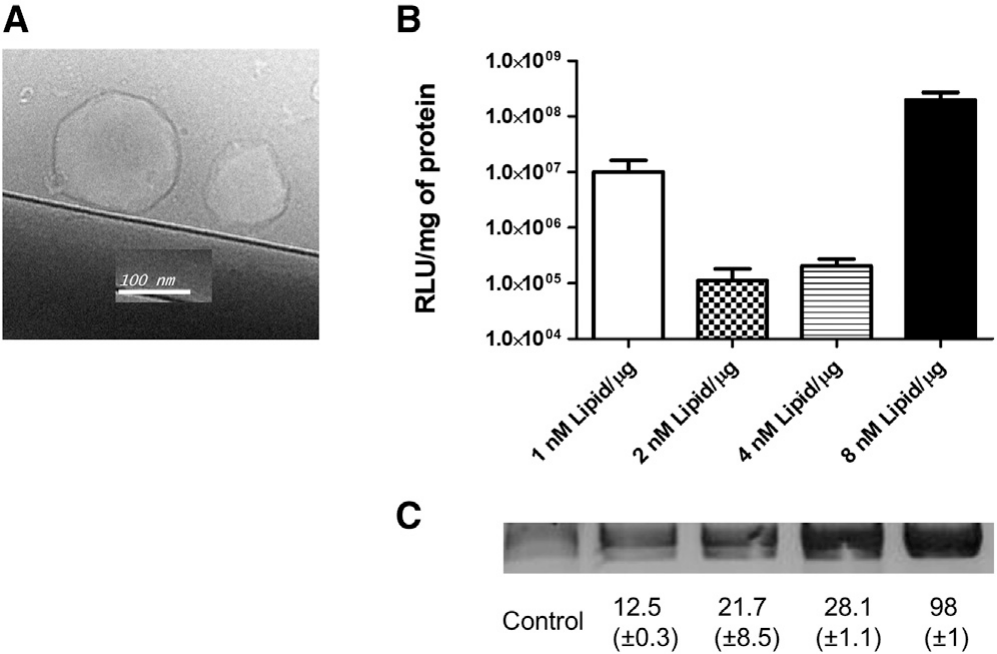
Effective Encapsulation of Plasmids into Liposomes (A) Cryoelectron microscopic picture of liposomes after the plasmid-loading step. In (B), results for the titration of encapsulating lipids versus DNA are shown. Murine melanoma cells (B16-F10) were transfected with different lipid/pcDNA3-luc formulations (experiments in duplicate; three independent experiments), and relative light units were measured 2 days after exposure of lipid/DNA complexes to cells. Error bars indicate SD. In (C), overnight DNase1 digests were conducted to elucidate the packaging efficiency in dependence of lipid/DNA ratios of the formulations tested in (B). The experiment was conducted using lipid/DNA formulations starting with initially 25 μg DNA packaged in the given lipid amounts, divided in three tubes, and digested with DNase1. Afterward, lipid/DNA formulations were chloroform extracted as described, and the aqueous fraction was loaded on 1% ethidium-bromide-stained TAE agarose gels. Below the gel, the relative density values of three gels measured by densitometry against the control lane, which contained 1 μg pcDNA3-Luc DNA (100% equals approximately 8.3 μg), are shown. Note that formulation with 8 nM lipids per μg DNA resulted in almost complete DNase1 protection of packaged DNA.

**Figure 2:**
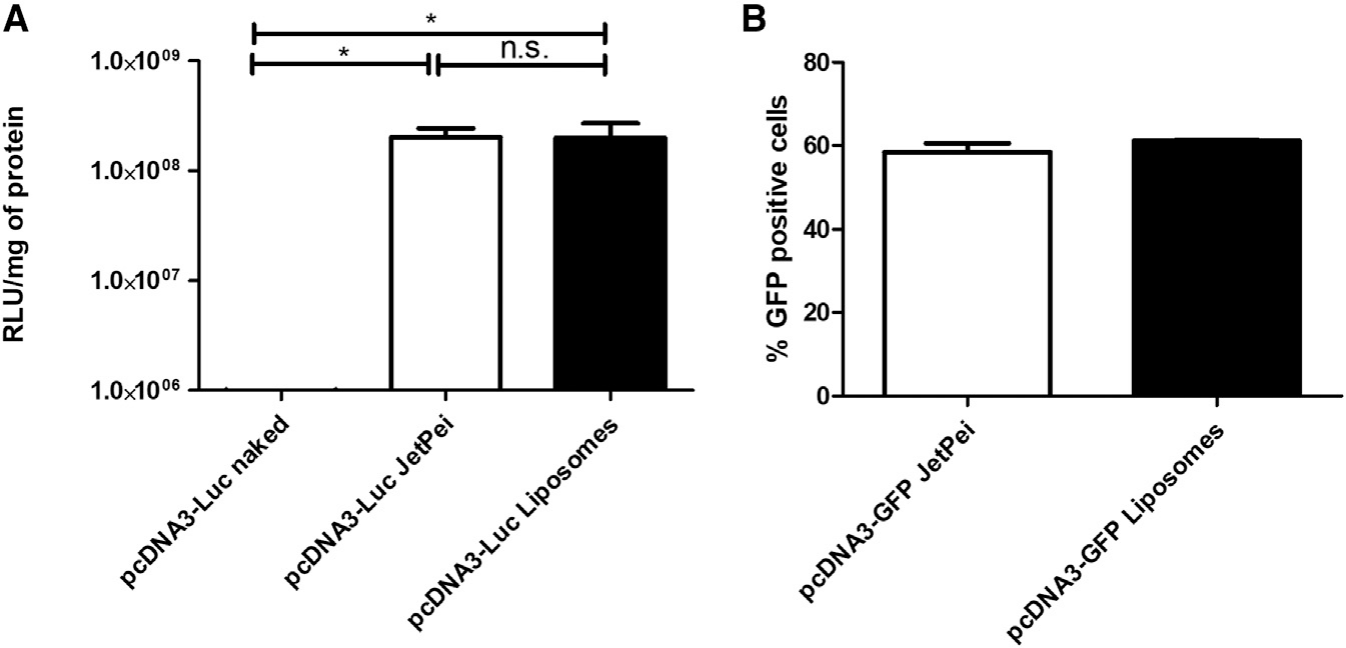
High Efficiency of Transfection Using Plasmid-Loaded Liposomes Compared to a Commercial Kit In (A), 5 × 10^5^ CHO-K1 cells were seeded in 6-well plates and transfected 24 hr later with carrier-free pcDNA3-Luc, associated to JetPei reagent, or loaded into liposomes. After 48 hr, cells were recovered and lysed, and relative light units per mg protein were measured in a luminometer. In (B), the plasmid pcDNA3-GFP was transfected in a similar way in HEK293T cells but without the control transfection with naked DNA. Forty-eight hours after transfection, cells were detached using PBS/0.05 M EDTA, and green fluorescence was measured by cytometry as described. Mock-transfected cells did not produce any discernible luciferase activity or presence of GFP under these conditions (data not shown). Both experiments were done in triplicate. Error bars indicate SD.

Different expression plasmids were cloned (Figure S1) and grown out to maxipreparations.^13^ The dynamics of plasmid association with liposomes is shown in Figure 1. After co-incubation with plasmid, a high percentage of plasmids associated to liposomes and were DNase1 protected. As expected, no signal was observed when unpackaged plasmids were digested in parallel with DNase1 (data not shown). Also, when DNA-loaded, non-extracted liposomes were loaded directly on agarose gels, no staining could be observed, indicating that confined plasmids are completely protected from the external environment and that DNA-intercalating reagents do not pass the liposome coat used herein (data not shown). The final size and appearance of DNA-loaded particles are shown in Figure 1A. The sizes obtained by Cryo-Stem differed slightly from those obtained by dynamic light scattering measurement (Table 1). To estimate transfection efficiencies of DNA-loaded particles in different cell lines, we first produced particles loaded with pcDNA3-luc and exposed the B16-F10 tumor cell line to formulations with the increasing lipid/plasmid coefficients. As shown in Figure 1B, substantial luciferase production was observed using either 1 nM lipids per μg DNA or 8 nM lipids per μg plasmid, while 2 and 4 nM/μg were less effective formulations. This test was done in triplicate and repeated once. Next, we compared the transfection efficiency with a commercial transfection reagent (JetPEI) in two other frequently used cell lines. As shown in Figure 2, similar amounts of luciferase activity were observed in DNA-loaded nanoparticle-transfected and conventionally transfected CHO-K1 cells (Figure 2A). Importantly, no specific steps, such as medium change, centrifugation, washing steps, or other, were applied during the liposome transfection procedure. These are often necessary using commercial kits or classical methods such as CaPO_4_ or DEAE-Dextran transfection. In another experiment, we also transfected the human cell line HEK293T with similar constructs using GFP instead of luciferase. After 2 days, almost 60% of the cells presented green fluorescence in both loaded liposome or JetPEI-transfected HEK293T cells (Figure 2B). This observation shows that DNA-loaded liposomes result in transfection efficiencies comparable to commercial kits in a number of different eukaryotic cells.

**Table 1:** Sizes, Polydispersity Indices, and ζ-Potential of Liposomes Before and After Encapsulation of DNA

Liposome Formulation	Size (nm)	Polydispersity Index	ζ-Potential (mV)
Liposome alone	77 ± 18.80	0.30 ± 0.0	–
Liposome + DNA	330 ± 80	0.80 ± 0.5	15 ± 4.6

### Immunizations with DNA-Loaded Cationic Liposomes Can Induce Antibodies against *Plasmodium vivax* CSP and MSP1_19_

Since the herein-used lipids are non-toxic,^14^ we asked if DNA-loaded liposomes could be used to induce a humoral response against many different antigens. For this, we used coding regions of the vaccine candidates *P. vivax* CSP (repeat region) and PvMSP1_19_. In order to enhance the immunogenicity of the corresponding proteins, gene fragments were cloned in frame with the gene for the small HBV antigen (HBsAg). Importantly, the PvCS-HBsAg fusion is reminiscent of the RTS/S construct and an integral part of the most clinically advanced human malaria vaccine to date.^15^

**Figure 3:**
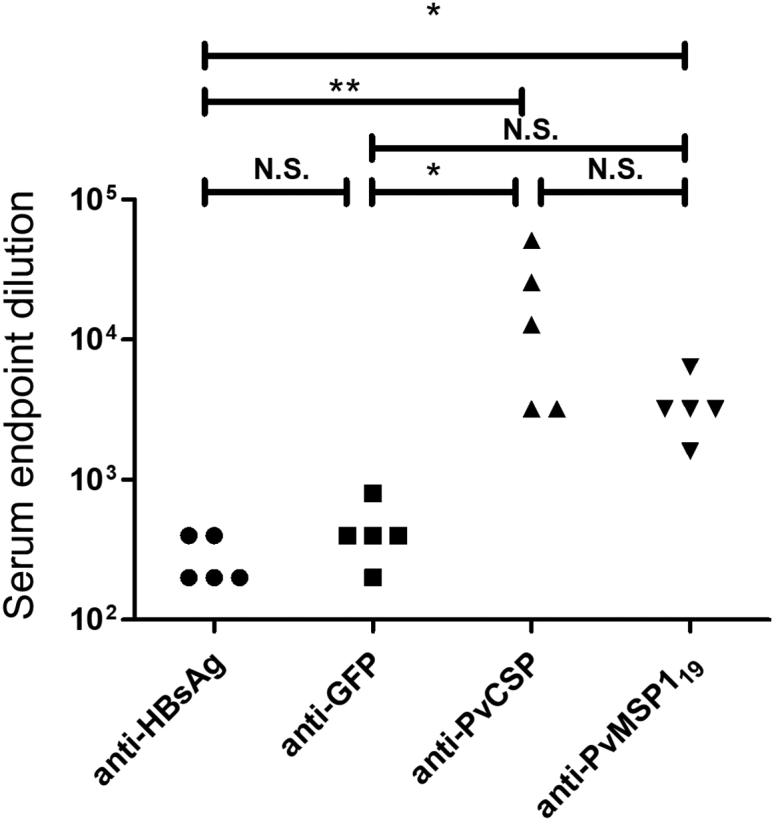
Liposome-Mediated DNA Immunization Results in Antibodies against the Fused Protein but Fewer against HBsAg Endpoint titers of anti-HBsAg, anti-GFP, anti-PvCS, and anti-PvMSP1_19_ containing sera were measured. Individual sera of 4 (GFP) or 5 liposome-packaged DNA-immunized mice were tested in endpoint dilutions, and the dilution factor where the sera still showed positive signals above background are shown. For ELISA plate coating, recombinant HBsAg, GST-GFP, GST-PvCS (repeat region), and GST-PvMSP1_19_ were used. Significantly different values are indicated by * or ** (p < 0.05 or p < 0.001, one-way ANOVA with Dunn’s multiple comparison test). N.S., not significant.

As controls, vectors encoding HBsAg alone and GFP without secretion signal were used. The DNA vaccine plasmids were entrapped in cationic liposomes at 8 nM lipids/μg and administered intraperitoneally in a quantity of 10 μg (before encapsulation) per animal. After four doses, the animals were bled and the sera tested for antibodies against PvCS, PvMSP1_19_, and HBsAg. Additionally, the content of anti-HBs was tested in western blots using GMP-grade recombinant HBsAg as target protein (Figure S2). After ELISA assays, we observed significant titers against recombinant PvCSP and PvMSP1_19_ with average values of 1:19,200 and 1:3,520, respectively. Lower titers were observed for GFP-specific antibodies (1:450) (Figure 3).

Although anti-HBsAg was only detected in mice that received the HBsAg encoding vector, these titers were quite low (Figure 3). Of note, low anti-HBs titers are a frequent problem in HBV vaccinees.^16^ On the other hand and in accordance to previous studies, these results show that the inclusion of HBsAg can greatly enhance the immune response against antigens that do not include T helper cell epitopes,^17^ such as PvMSP1_19_.^18^

### Cationic Liposome-Encapsulated Plasmid DNA Can Produce Antibodies against PfRH5 with Minor Recognition of HBsAg

**Figure 4:**
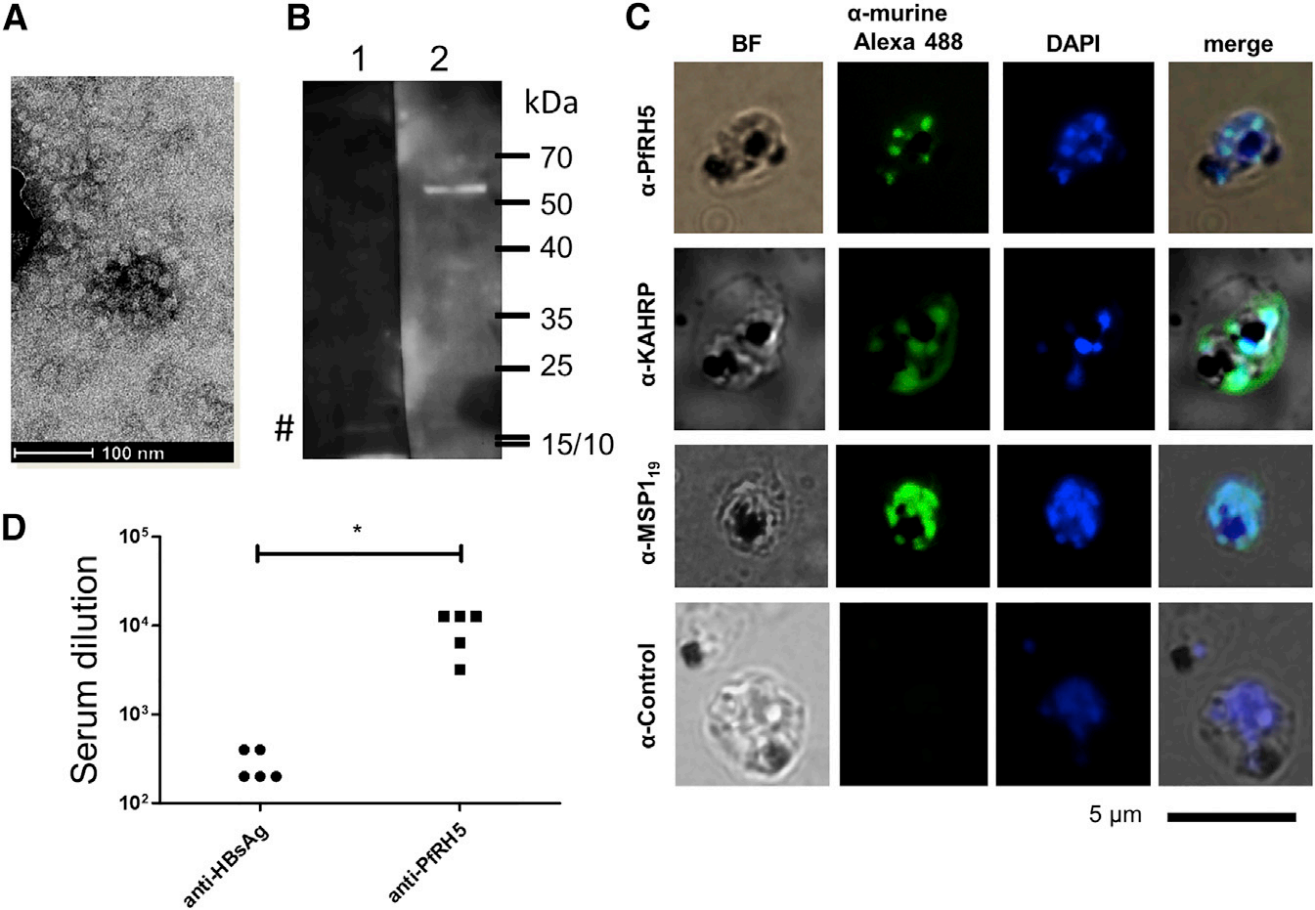
Sera from pcDNA3-PfRH5-HBsAg Immunized Mice Recognize Native PfRH5 in *P. falciparum* NF54 Schizont Extracts In (A), material from a sucrose gradient-floated supernatant from PfRH5-HBs-producing cells (25%–30% fraction) is visualized by electron microscopy. In (B), antiPfRH5 from immunized mice recognizes a protein species coincident with processed PfRH5 (lane 2), while pre-immune serum does not (lane 1). Note the unspecific reaction of a 16-kDa protein with both sera (#). In immunofluorescence microscopy, pooled immune sera (1:20) also recognize PfRH5 in native late-stage schizonts (C, upper panel), similarly to antiMSP1_19_ (third panel), both associated to merozoites. A polyclonal anti-KAHRP serum recognized infected red blood cell (IRBC)-surface located and cytoplasmic proteins. No fluorescence was observed using pre-immune serum (C, bottom panels). (C, left panels) IRBC visualized in bright field (BF), (second panels from the left) reacting with specific antibodies and detected with anti-mouse Alexa 488, (second panels from the right) nuclear staining with DAPI, and (right panels) overlay of the images on the left. In (D), the endpoint dilution ELISA showed antibody titers against GST-PfRH5 comparable to those obtained against PvCS and PvMSP1_19_, while the obtained titers against the HBs domain were lower and similar to the previous experiment. No significant titers against PfRH5 or HBsAg were found in pre-immune sera (data not shown).

In the next step, we asked if the fusion of one of the most promising malaria vaccine candidates to date, PfRH5, to HBsAg would also result in the production of antibodies against the corresponding protein. Using a plasmid encoding PfRH5 fused with HBsAg, we first tested if the PfRH5-HBsAg construct administered intramuscularly as naked DNA elicited antibodies against PfRH5. This was not the case (data not shown). Then, PfRH5-HBsAg encoding plasmid DNA was encapsulated as before and immunized. In parallel, the same plasmid was also transfected in HEK293T cells, and the supernatants of the culture were fractioned via 10%–60% sucrose gradients in PBS in order to concentrate eventual subviral particles in the 25%–30% fraction. As shown in Figure 4A, virus-like particles of roughly 20–30 nm could be observed in transmission electron microscopy, and the size coincides with the size determined by dynamic light scattering (DLS) (Figure S3). We then looked for the generation of PfRH5-specific antibodies (Figure 4B). First, immune sera recognized one single protein species in parasite lysates, coincident with the size of single processed PfRH5^19^ of approximately 53 kDa, while antibodies in the pre-immune sera did not visibly react. Second, immune sera were able to recognize merozoites in immunofluorescence assays showing a punctuate pattern as expected for proteins localized in rhoptries (Figure 4C). In the same experiment, anti-KAHRP identified cytoplasmic and infected red blood cell surface-bound KAHRP while anti-MSP1_19_ antisera recognized merozoites. We also monitored the antibody endpoint titers achieved with this construct, and the observed titers against GST-PfRH5 were in the order of 10^4^ (Figure 4D). Only very low anti-HBs titers were observed in mice immunized with PfRH5-HBsAg-encoding plasmids (10^2^ for anti-HBs; Figure 4D).

### Antibodies against PfRH5 Can Block *P. falciparum* Reinvasion In Vitro in a Dose-Dependent Manner

**Figure 5:**
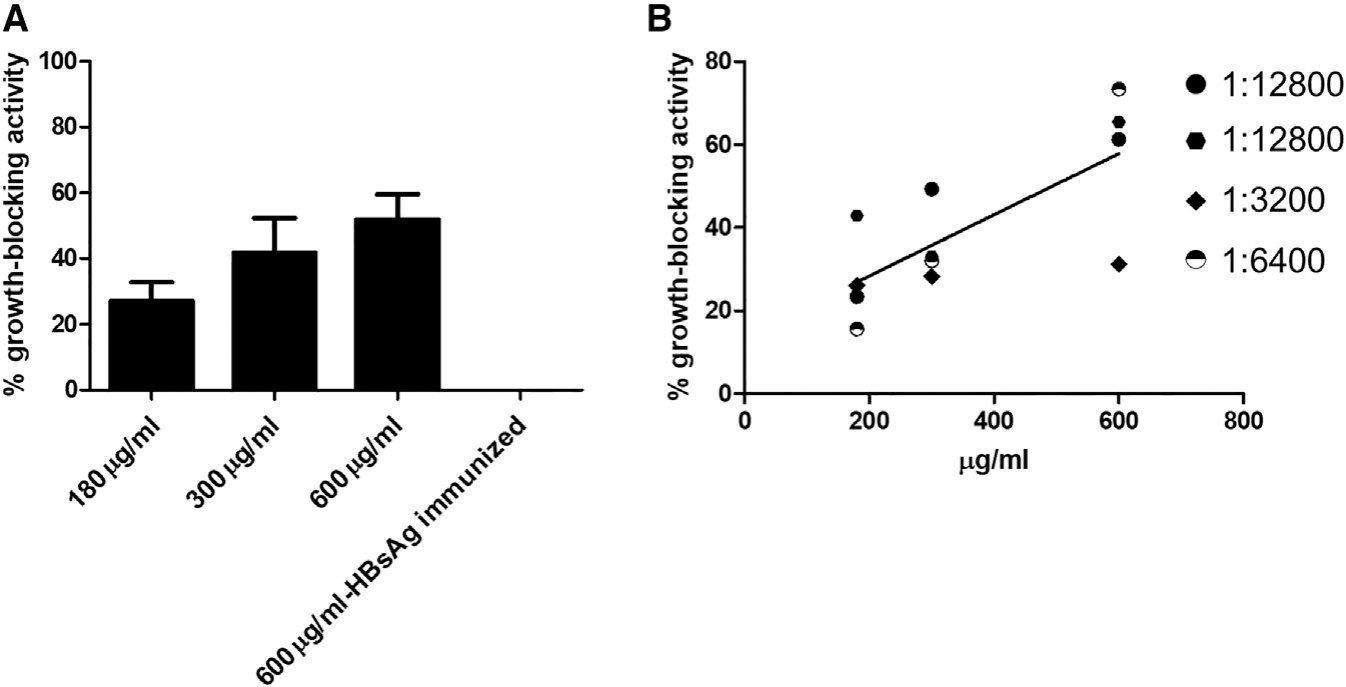
Purified IgGs from Liposome-Encapsulated pcDNA3-PfRH5-HBs-Immunized Mice Inhibit *P. falciparum* Red Blood Cell Reinvasion In (A), the percentage of growth inhibition of parasite proliferation (measured by flow cytometry) is shown for three dilutions of purified IgGs from four mice immunized with encapsulated PfRH5-HBs encoding vector as described. The shown values were calculated by comparing parasitemias in parallel cultures supplemented with 600 μg/mL non-related IgG (=0% growth inhibition). Note that IgG from pcDNA3-HBs-immunized mice did not inhibit parasite proliferation. In (B), the dose dependency is demonstrated by plotting the content of antiPfRH5-specific titers against the growth inhibitory/blocking effect of IgG. Data were normalized and analyzed by Pearson’s correlation (r^2^ = 0.54, p = 0.006). Each symbol means the values obtained by purified IgG from four different mice with the indicated anti-PfRH5 endpoint titers. Error bars indicate SD.

An important landmark in the quality of anti-malarial antibodies generated through vaccination is their inhibitory effect in growth- or reinvasion assays. In order to evaluate this effect and compare it with previous studies,^11^ the immunoglobulin G (IgG) fractions of immune sera were protein A purified and added in different concentrations to *P. falciparum* in vitro cultures. As shown in Figure 5A, purified IgGs were able to inhibit parasite growth in a dose-dependent manner. As expected, the IgGs from mice with different anti-PfRH5 titers showed a titer-dependent relation with their blocking activity (Figure 5B; r^2^ = 0.54, p = 0.006, linear regression). This indicates that the PfRH5-HBsAg encapsulated DNA vaccine formulation is efficient in inducing functional antibodies with an affinity sufficiently high enough to decrease merozoite invasion, perhaps by interfering in the PfRH5-Basigin interaction. Of note, purified IgG from mice immunized with HBsAg-coding plasmids did not exert any reinvasion/proliferation blocking effect (Figure 5A).

### Encapsulated DNA Immunization Induces a Specific Cellular Response

**Figure 6:**
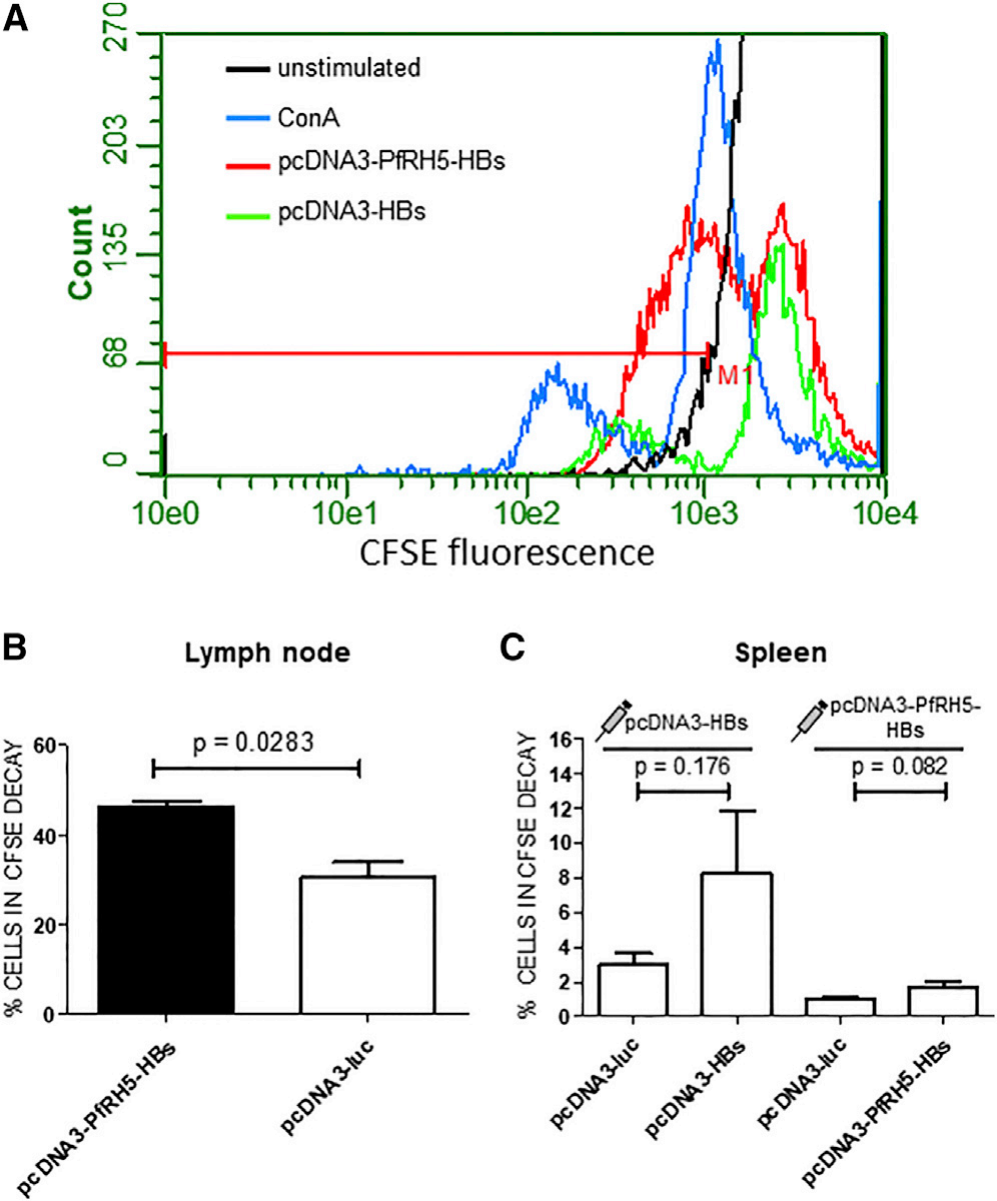
Inguinal Lymphocytes from DNA-Loaded Liposome-Immunized BALB/c Mice Are Stimulated by Specific Antigens Lymphocytes from spleen or inguinal lymph nodes were removed from immunized mice and exposed to different stimuli in vitro. Proliferation assays took place 60 days after the last immunization with either liposome-packaged pcDNA3A-PfRH5-HBs or pcDNA3A-HBs. 5 × 10^5^ cells were labeled with 100 ng of CFSE and then split. Cells were then challenged by exposure to DNA-liposome formulations (1 μg/mL of DNA per 5 × 10^5^ cells) that were used in the immunizations (plasmids pcDNA3-PfRH5-HBs and pcDNA3-HBs). As a control, a non-specific reporter plasmid (pcDNA3-Luc) was used. After 3 days (72 h), fluorescence levels in cells were analyzed by flow cytometry. In (A), the exemplary cytometer readouts of the CFSE decay from inguinal lymph node cells of one single mouse stained with CFSE is shown. Cells were stimulated with Concanavalin A (blue), encapsulated pcDNA3-PfRH5-HBs (red), and encapsulated pcDNA3-HBs (green). The signal from unstimulated cells is shown in black. Note that an excess of unstimulated cells is shown (cut peak) and no smaller peaks of dividing unstimulated cells are visible. While the percentage of dividing cells was highest in Con-A-stimulated cells (shift of highly fluorescent cells on the right to less fluorescent—divided—cells on the left), fewer divisions were observed in pcDNA3-PfRH5-HBs-stimulated cells, and still fewer divisions in pcDNA3-HBs-stimulated cells were recorded. The fluorescence limit of non-dividing cells is marked by M1, and peaks left from this point were from divided cells. In (B), the CFSE decay in inguinal lymph node cells from mice immunized with pcDNA3-PfRH5-HBs and stimulated with either liposome-loaded pcDNA3-PfRH5-HBs or pcDNA3-luc (unrelated control) is shown. Statistical analysis was performed using a paired t test with n = 4 animals. In (C), the proliferation rate from spleen cells from mice immunized with pcDNA3-HBs (left two bars) and pcDNA3-PfRH5-HBs (right two bars) and stimulated with the same plasmids packaged in liposomes and the respective control with pcDNA3-luc loaded liposomes is shown. Statistic tests were done as in (B) (n = 4 animals). See Supplemental Information for cytometer settings. Error bars indicate SD.

The delivery of DNA vaccines into cells causes the production of proteins and subsequent presentation in major histocompatibility I (MHC I) complexes, which then stimulate T cell responses. To test if this was the case in the herein-used application of DNA delivered in liposomes, BALB/c mice were immunized with three doses of pcDNA3-PfRH5-HBs and had their lymphocytes removed from either inguinal lymph nodes or the spleen. Then, these lymphocytes were stimulated with liposomes packaged with pcDNA3-Luciferase, pcDNA3-PfRH5-HBs, or pcDNA3-HBs. Afterward, cell proliferation was monitored by 5(6)-carboxyfluorescein N-hydroxysuccinimidyl ester (CFSE) assays (Figure 6A). As a result, baseline signals were observed when lymphocytes were stimulated with pcDNA3-Luciferase, which leads to the production of the unrelated Luciferase protein (Figures 6B and 6C). In contrast, significant stimulation was observed when cells from inguinal lymphocytes were stimulated with pcDNA3-PfRH5-HBs (Figure 6B). Since few cells were recovered from lymph nodes, we did not test the stimulation of cells from mice immunized with pcDNA3-PfRH5-HBs using liposomes packaged with pcDNA3-HBs, which would show if the reaction observed was directed against HBs or the PfRH5 domain. A proliferative response, yet much weaker, was also seen in cells from the spleen (Figure 6C). Interestingly, spleen cells from mice immunized and stimulated with pcDNA3-HBs showed a stronger reaction than cells from mice immunized and stimulated with pcDNA3-PfRH5-HBs. In any case, the difference in proliferation between pcDNA3-luc and pcDNA3-HBs or pcDNA3-PfRH5-HBs was not statistically significant. Interestingly, the lymphocytes from lymph nodes seem more sensitized to stimulation with plasmid DNA, since around 30% of these cells underwent proliferation with pcDNA3-luc, while spleen cells were only weakly stimulated, and around 3% showed a proliferative response. This result indicates that the immunization with plasmid DNA entrapped in liposomes can create a cellular immune response. The inclusion of a murine γ-interferon-expressing vector may have enhanced this response. In another experiment, we asked which subset of lymphocytes could be stimulated upon contact with encapsulated plasmids. For this, mice were immunized with pcDNA3-PfRH5-HBs as described, and their splenocytes stimulated with encapsulated pcDNA3-luc (unrelated control), pcDNA3-HBs, or pcDNA3-PfRH5-HBs. Then, different subsets were detected with antibodies against CD4, CD8, and CD19. While only a basal labeling could be observed with pcDNA3-luc, a significant survival of CD19^+^ cells was observed through contact with encapsulated pcDNA3-HBs and pcDNA3-PfRH5-Hbs, and a significant survival of CD8^+^ cells was also detected after stimulation with encapsulated pcDNA3-PfRH5-HBs (Figures S4 and S5). This means that there are antigen-reactive B (CD19^+^) and T (CD8^+^) cells and perhaps CD4^+^ T cells after encapsulated-plasmid immunization, although antigen-reactive CD4^+^ T cells were not significantly increased.

## Discussion

Recently, the European health agency endorsed the use of the first malaria vaccine RTS/S.^15^ Although the vaccine probably helps to save lives and significantly decreases the incidence of clinical malaria and the number of severe malaria episodes in vaccinees,^20^ it still does not achieve protection rates observed for common antiviral vaccines such as influenza or HBV vaccines. Other malaria-vaccine approaches to date were less successful and still did not provide sufficient protection (for a review, see Birkett^21^) or may be difficult to deliver in field situations.^22^ On the other hand, recently discovered structures, such as PfRH5^11^ or the proteins RIPR and CYRPA, with which it forms a complex,^23^ are very promising but difficult to produce in recombinant form. While approaches exist that use native antigens as effective immunogens,^24^ these are challenging to be scaled up for a broader use. For this reason, we envisioned that the immunization of promising, hard-to-produce vaccine targets may be viable when administered as a DNA vaccine. However, the delivery of a DNA vaccine still poses the problem of low efficiency. This disadvantage can be overcome when using DNA encapsulated in fusogenic liposomes of nanometric size using microneedle devices in the example of an HBV^25^ or HIV^26^ immunization and/or proper formulation of encapsulating lipids.^27^

Herein, we formulated cationic liposomes made from three biocompatible lipids and encapsulated plasmid vectors expressing three well-studied malaria-vaccine targets as well as reporter genes eGFP and luciferase. When measuring the transfection efficiency of plasmid-loaded liposomes in in vitro experiments, we observed the successful transfection of more than 50% of the plasmid-liposome exposed cells, similar to the efficiency of a commercial reagent tested in parallel. This value is much higher than the transfection rate achieved using another system of DNA-loaded liposomes made from archae-membrane lipids (archaeosomes^28^). Given the biocompatibility of the herein-used lipids, we asked if the plasmid-loaded liposomes would also induce the production of proteins in whole organisms such as BALB/c mice.

Considering that a high surface density of proteins can enhance their immunogenicity, we genetically fused a number of antigens to the small HBV envelope antigen, which itself forms the so-called 20 nm particles. The improved immunogenicity of antigens displayed on 20 nm particles was shown for a couple of examples (e.g., the RTS,S vaccine, *P. vivax* MSP1_19_,^17^ and poliovirus envelope proteins^29^). Importantly, even the 50 kDa fusion of PfRH5 to HBsAg seemed to elicit the production of 20–25 nm particles in transfected cells as detected by dynamic light scattering analysis (Figure S3). When directly comparing the obtained antibody titers against PvMSP1_19_, we did not observe significantly higher titers using the liposomal approach compared to a study using the construct as a naked, intramuscularly delivered DNA vaccine^30^. Nevertheless, the herein achieved titers were much higher compared to the genetic vaccination of either CS^31^ or MSP1_19_ alone,^32^ underlining the enhancing effect of HBsAg in the construct. However, it is unclear to what degree the secretion signal tissue plasminogen activator (TPA) included in our constructs influenced the antibody response. In an earlier study, the inclusion of the TPA secretion signal in DNA vaccines using *Plasmodium yoelii* merozoite protein 1 gene fragments was not advantegous,^32^ while the delivery of *P. falciparum* MSP4 as a DNA vaccine was more efficient when delivered as a TPA-tagged construct.^33^

Importantly, the doses used for immunization herein were much smaller, and the uniformity in antibody titers in individual mice was much improved.^30^ Liposomal DNA vaccination involving HBsAg is supposed to stimulate antigen presentation by primary dendritic cells in both MHC1 and MHC2 receptors.^34^ Although not tested for, it is therefore expected that antigen-specific T and B cells, including memory cells, are developed and that immune response can be boosted upon contact with the native antigens during infection. Additionally, the proliferation of cells from pcDNA3-HBs- or pcDNA3-PfRH5-HBs-immunized mice after stimulation with pcDNA3-HBs-encoding plasmids indicates that a cell-based response was generated against HB peptides (see Figure S5), even though only modest anti-HB titers could be observed.

The application of naked DNA vaccines commonly targets skin-resident antigen-presenting cells such as keratinocytes, Langerhans cells, and others.^35^ Herein, the intraperitoneal route of immunization was used—which is easy to apply but inefficient when using naked DNA. In support of the hypothesis that injected DNA-loaded liposomes are readily taken up by peritoneal cells, we detected most antigen-reactive lymphocytes in inguinal lymph nodes. Lymphocytes drained to the spleen responded less to stimulation with either liposome-packaged plasmid, indicating that the main response and antigen presentation occurs in lymph nodes. On the other hand, the overall proliferative response in lymph node cells compared to spleen cells was around 10 times stronger using the control plasmids, possibly reflecting a different distribution of cell subsets in both organs. When testing which cells were mainly primed by encapsulated DNA immunization, we observed that CD19^+^ (B-lymphocytes) and CD8^+^ (T-lymphocytes) lymphocytes had their survival extended, meaning that these cells were effectively stimulated by splenocytes presenting the plasmid-encoded antigens against which was immunized (Figures S4 and S5). In order to avoid unspecific retention and to produce a stronger transfection effect, we did not apply any commonly used adjuvant such as Alum to the particles, since this seems deleterious when using DNA vaccines.^36^ However, in one case we tried to enhance the immune response by co-encapsulating a γ-interferon-encoding plasmid into liposomes. In a related model, this co-administration increased the response against Woodchuck HBV core protein delivered as a naked DNA vaccine.^37^

For the use in vaccine applications, the route of delivery of DNA-loaded liposomes has to be modified. A minimally invasive way of delivery would be via skin patches, such as that shown for the application of ceramic microneedles^38^ or via the intranasal route.^39^ Specifically, the intranasal delivery of *Mycobacterium tuberculosae* hsp86-encoding DNA seemed to induce a systemic Th1-type response. Although never specifically shown, a Th1 response reacting against infected liver cells may be desirable in the case of *Plasmodium* sporozoite infection where the effective reduction of infected liver cells is supposed to limit the development of blood stage malaria (for a review, see Hill^40^). Although mucosal immunization is more likely to induce an IgA type of response, a systemic response including CD8^+^ and CD4^+^ cells can also be developed (reviewed in Tawari et al.^41^).

To prove the efficiency of the immunization, we tested if antibodies generated against a promising vaccine candidate—PfRH5—were able to reduce reinfection of erythrocytes in in vitro growth assays. It was expected that the antibodies that were able to recognize native PfRH5 in western blots would also exert a reinvasion-blocking activity. As shown above, this was the case, and the values for the inhibitory activity were comparable to what was achieved after recombinant adenovirus-based approaches^11^ or proteoliposome-mediated immunization.^24^ For example, in the study by Douglas and colleagues,^11^ anti-PfRH5 titers ranging from 10^4^–10^5^ were achieved through immunization of rabbits with recombinant adenovirus. Growth-inhibition assays (GIAs) showed around 40% inhibition when using the purified IgG fraction at 625 μg/mL. In another report, where bacterially produced and refolded PfRH5 was produced and immunized,^42^ titers of >10^5^ were obtained. However, purified IgG at comparable concentrations of 500 μg/mL or 1 mg/mL in GIA assays showed maximally around 40% growth inhibition in the *P. falciparum* HB3 strain, whereas the inhibition observed for the 3D7 strain (clone of the herein-used NF54 isolate) was below 20%.^42^ In our study, purified mouse IgG after immunization reacted equally in immunofluorescence assays and also showed a very comparable result of around 50% inhibition at 600 μg/mL purified mouse IgG. This result shows that bacterially produced PfRH5 may not be as efficient in creating antibodies with blocking activity than PfRH5 produced in mammalian cells. Another approach modified the PfRH5 protein and used *Drosophila* cells for expression.^43^ In this study, sera generated by immunization with the best performing construct achieved slightly weaker *P. falciparum* growth inhibition compared to adenovirus or DNA-liposome-mediated immunization (50% growth inhibition at ∼1.2 mg/mL IgG). GIA experiments performed herein or done by several other groups used purified IgG. In this context, it must be considered that under natural conditions, complement may still further enhance the protective effect already observed by using purified IgG as shown by Boyle and colleagues.^44^ Importantly, adenovirus and plasmid-DNA-loaded liposome approaches function by the fact that the vaccine antigen is ultimately synthesized by the vaccinee’s cells, thus permitting the synthesis of a perfectly folded protein. We envision that the immunization of a mixture of plasmids encoding separately the proteins CYRPA, RIPR, and PfRH5, which together form the red blood cell invasion complex,^23^ may even produce better results than the usage of only PfRH5.

Taken together, we presented an approach where cationic liposomes were successfully loaded with plasmid DNA and showed that the injection led to a significant and multileveled (antibody and cell-mediated) response. Ideally, different plasmids can be deliberately mixed and encapsulated, underlining the versatility of this approach. Using simultaneously proteic ligands on the surface of the DNA-loaded liposomes, it still may be possible to further influence/enhane the overall effect by directing liposomes to specific cells.

## Materials and Methods

### Liposome Preparation and Entrapment of Plasmid DNA

If not otherwise stated, all chemicals were from Sigma-Aldrich/Merck or Carl Roth (Darmstadt, Germany). All lipids used herein were from Avanti Polar Lipids (Alabaster, Alabama, USA). Liposomes were prepared using DDAB (dimethyl-di-octadecyl-ammonium), cholesterol (molar ratio 4:1), and DSPE+PEG2000 (1,2-distearoyl-*sn*-glycero-3-phosphoethanolamine-N-[amino(polyethylene glycol)-2000], 5% of total lipids used). Initially, DDAB, cholesterol, and DSPE-PEG2000 were dissolved in 1 mL of chloroform and were then left under a constant N_2_ flow for evaporation of chloroform and the formation of a phospholipid film on the tube walls. This film was then maintained under vacuum for at least 1 hr to remove any remaining chloroform. The film was rehydrated in 5 mM Tris-HCl (pH 7.5) at 60°C (DDAB transition temperature) for 1 hr and vigorously stirred every 10 min. The obtained opaque solution was then subjected to sonication at high energy until the solution became almost completely transparent. Then, the solution was centrifuged for 1 hr at 100,000 × *g*. Any remaining pellet was discarded, and the supernatant containing unilamellar lipid vesicles were used for preparation of the liposomal formulation.

The cationic liposomal nanoparticles were loaded with target DNA using different amounts of plasmid per nmol of lipid (from 1 nM to 8 nM DDAB lipids per μg plasmid). Then, seven alternating cycles of freezing in liquid nitrogen for 1 min and thawing in a water bath of 60°C for 1 min were applied. Following this, the formulation was passed through polystyrene membranes with pore sizes of 0.1 μm for at least seven times (extrusion). All plasmid DNA constructs used herein are shown in Figure S1.

### Retention Test

To test retention in liposomes, plasmids were encapsulated in liposomes in different lipid-to-plasmid quantity ratios as described. After entrapment, DNase I (Fermentas, Thermo Fisher Scientific) was added to each formulation, including a control without cationic liposomes. The product of this digestion was extracted by phenol/chloroform and centrifuged, and ethanol and Na-acetate (pH 5.2, 0.3 M final) was added for precipitation on ice for 1 hr. After centrifugation at 10,000 × *g*, the extraction product was submitted to electrophoresis in the presence of ethidium bromide in a conventional TAE agarose gel of 1%. As an additional control, liposomal DNA vaccines without phenol extraction and plasmid alone without any manipulation were used as a standard to visualize plasmids that could be protected from DNase treatment.

### Immunization

Five groups of five male BALB/c mice (6 weeks old) were immunized intraperitoneally in accordance with national animal welfare regulations (Protocol Number 15_03_03 from 2013; approval emitted by the Board for Ethics in Animal Experimentation at the Institute for Biomedical Sciences, USP). We used 4 doses of 10 μg DNA vaccine/cationic particles to immunize animals every 15 days. In order to enhance immune reaction, the last dose was performed in the presence of a pcDNA3-based plasmid expressing murine interferon-gamma.^45^ This plasmid was used in a ratio of 1:100 relative to the antigen-encoding plasmid. Blood was collected on days 0 (pre-immune serum), 14, 28, and 42 and after 66 days.

### ELISA and Western Blots

Each 96-well ELISA medium-binding plate (Jet Biofil, Guangzhou, China) was coated with the antigen at 200 ng/well at 4°C overnight. On the next day, plates were washed with PBS/Tween 0.05% (PBS/T) and blocked with 2% skimmed milk/PBS for 1 hr at room temperature. Plates were washed and incubated with (1) antisera generated in mice immunized with pcDNA3-PfRH5-HBs and antibodies generated in mice immunized with pcDNA3A-HBs for plates coated with purified GMP-grade HBsAg (a gift from Butantan Institute, São Paulo, Brazil), (2) antisera generated in mice immunized with pcDNA3-PvCS-HBs using the repeat region of the circumsporozoite gene of *P. vivax* (Salvador variant), or (3) animal sera immunized with pcDNA3-PvMSP1_19_-HBs^17^ for coating with PvMSP1_19_. Finally, antisera from mice immunized with the control plasmid pcDNA3-GFP were analyzed using plates coated with GFP (see Figure S1 for a map of all plasmids used). Antisera were endpoint-diluted and incubated for 2 hr at room temperature. After four washing steps with PBS/T, an anti-murine-IgG coupled to horseradish peroxydase (KPL-Seracare, Milford, MA, USA) was applied for 1 hr at room temperature (1:2,000). After repeated washings, all wells were developed with 3,3’,5,5’ tetra methyl benzidine (TMB) substrate (Pierce, Thermo Fisher Scientific) and stopped after 5 min with 1 M HCl, and the result of the colorimetric reaction was analyzed in a BioTek plate reader (BioTek, Winooski, VT, USA) at 450 nm/595nm.

For western blot, 5 μg HBsAg were electrophoresed under non-reducing conditions in SDS-polyacrylamide gels (10%). After this, proteins in gels were transferred onto nitrocellulose membranes (Hybond C, GE Healthcare, São Paulo, Brazil), and these were blocked with 2% milk in PBS/T for 1 hr at room temperature. After two washing steps with PBS/T, primary antibodies (serum pool of plasmid-liposome-immunized animals) were incubated for 1 hr at room temperature at a dilution of 1:500 in 1% milk PBS/T. After five washes with PBS/T, membranes were incubated with horseradish peroxidase (HRP)-conjugated anti-mouse secondary antibody diluted 1: 2,000 for 1 hr at room temperature. After five washes with PBS/T, the membranes were briefly soaked in enhanced chemiluminescence (ECL) reagent (GE Healthcare), and chemiluminescence was documented on Hypermax X-ray films (Kodak) or photographed using a GE Image Quant 3000 apparatus.

### Transfection Efficiency Test In Vitro

To test the transfection efficiency of cationic liposomal nanoparticles loaded with DNA, a test plasmid expressing *Photinus pyralis* luciferase under the control of the immediate-early cytomegalovirus (CMV) promoter was used (pcDNA3-luc). For this, different eukaryotic cell lines (HEK293T/17, ATCC no. CRL-11268, CHO-K1, ATCC no. CCL-61, B16-F10, ATCC no. CRL-6475) were grown to semiconfluency and exposed to either 5 μg naked, non-complexed plasmid or 5 μg of pcDNA3-luc complexed to the transfection agent JetPei (Polyplus Transfection, Illkirch, France) or 5 μg pcDNA3-Luc encapsulated in cationic liposomes. Forty-eight hours after transfection, cells were washed and lysed, and the luciferase activity was measured using Bio-Rad luciferase reagents (Bio-Rad, Hercules, CA, USA). Relative light units were measured in a Berthold Lumat LB9 luminometer (Berthold Technologies, Bad Wildbad, Germany). The data were normalized for total protein against relative light units (RLUs). Experiments were conducted in triplicate.

### Cell Proliferation Assays

Proliferation assays took place 60 days after the last immunization, when spleens and inguinal lymph nodes of immunized mice were removed from animals that had been inoculated with either encapsulated pcDNA3A-PfRH5-HBs or pcDNA3A-HBs. 5 × 10^5^ cells were washed and cultured in RPMI medium supplemented with bicarbonate and 5% fetal calf serum at 37°C under a 5% CO_2_ atmosphere and subsequently labeled with 100 ng of CFSE (carboxyfluorescein succinimidyl ester; Becton Dickinson, São Paulo, Brazil). The cells were then challenged by exposure to liposome formulations that were used in the immunizations, that is, nanoparticles containing plasmids pcDNA3-PfRH5-HBs and pcDNA3-HBs. As a control, we used a non-specific reporter plasmid (pcDNA3-Luc) in the same liposome construction. After 3 days, fluorescence levels in cells were analyzed by flow cytometry (Guava Easycyte Cytometer, Merck-Millipore, Darmstadt, Germany). For detection of surface-marker expression, similar challenge experiments were conducted followed by antibody labeling using murine anti-CD4, -CD8, and -CD19 (antibodies from Becton Dickinson). Cytometer gating settings are shown in Figure S4.

### *P. falciparum* Culture and Invasion Inhibition Assays

The NF54 strain of *P. falciparum* was maintained in human B+ red blood cells (hematocrit 5%) in RPMI medium supplemented with 10% human B plasma or 0.5% Albumax 1 (Invitrogen/Thermo Fisher Scientific). Cultures were kept in candle jars in an incubator at 37°C with daily medium exchange.^46^ Parasitemias were monitored by thin blood smears stained with modified Giemsa stain (Panótico Quick kit, LaborClin, Pinhais, Brazil) and microscopy. Cultures were synchronized by intermittent plasmagel flotation^47^ (Voluven 6%; Fresenius-Kabi, Campinas, Brazil) followed by sorbitol lysis^48^ and plated in 96-well plates at an initial parasitemia of 1%. Protein-A-purified IgG fractions from immunized mice were added at different concentrations (0.5 μg/mL, 1 μg/mL, 3 μg/mL, and 5 μg/mL) and volumes were matched with RPMI medium. Measurements were taken after 24 hr and 48 hr. In order to measure the parasitemia, aliquots were removed from wells and stained with ethidium bromide and analyzed by flow cytometry (Guava easycyte) as described previously.^49^

### Immunofluorescence

Live parasite immunofluorescence assays were conducted with trophozoite/schizont-enriched *P. falciparum* using a protocol modified from Tonkin and colleagues.^50^ In brief, detecting IgG were from mice that were immunized with DNA vaccine/cationic liposomes. Control antisera anti-GST-PfMSP1_19_ and anti-GST-KAHRP were generated using recombinant GST-PfMSP1_19_ and GST-KAHRP as described elsewhere.^45^ Infected red blood cells in suspension were permeabilized with 0.01% Saponin/RPMI 3% fetal calf serum (FCS) for 30 min and incubated with a 1:500 dilution of anti-PfRH5 serum pool obtained from mice together with 40 μg/mL of 4′-6-diamidino-2-phenylindole (DAPI; Sigma-Aldrich/Merck, Darmstadt, Germany) for 30 min. After three washing steps with RPMI 3% FCS, an Alexa Fluor 488-labeled anti-mouse-IgG (Molecular Probes/Thermo Fisher Scientific, 1:1,000 dilution) was applied for 30 min. After another three washing steps, immunofluorescence was visualized on a Zeiss Axio Imager A2 fluorescence microscope (Carl Zeiss, Jena, Germany).

### Cryoelectron Microscopy

Liposomes were prepared as described above and analyzed by cryoelectron microscopy as previously published.^51^ For this, 3 μL DNA-loaded liposomes were spotted to a holey carbon-film grid (Quantifoil Micro Tools, Jena, Germany), previously pretreated with Gatan Solarus 950 plasma cleaner. Specimen-coated grids were plunge-frozen in liquid ethane using a Gatan Cryoplunge 3 (Gatan, Pleasanton, CA, USA). Low-dose imaging of the frozen, hydrated specimen kept in liquid nitrogen with a Gatan 626 single tilt cryoholder was performed on a JEM2100 electron microscope (JEOL, Tokyo, Japan, operating at 200 kV). A Gatan Ultrascan 4000 CCD camera was used to record images (∼40,000× magnification).

## Author Contributions

W.L.F., D.J.I., and G.W. conceived the experiments. W.L.F., R.S., and B.N.M.M. did the experimental work. W.L.F. and G.W. wrote the manuscript. D.J.I. provided material.

## Supplemental Information

Document S1. Figures S1–S5

Document S2. Article plus Supplemental Information

## References

[1] Naldini L. (2015). Gene therapy returns to centre stage. Nature.

[2] Saade F., Petrovsky N. (2012). Technologies for enhanced efficacy of DNA vaccines. Expert Rev. Vaccines.

[3] Gopal G.J., Kumar A. (2013). Strategies for the production of recombinant protein in *Escherichia coli*. Protein J..

[4] Gramzinski R.A., Millan C.L., Obaldia N., Hoffman S.L., Davis H.L. (1998). Immune response to a hepatitis B DNA vaccine in Aotus monkeys: a comparison of vaccine formulation, route, and method of administration. Mol. Med..

[5] Mingozzi F., High K.A. (2013). Immune responses to AAV vectors: overcoming barriers to successful gene therapy. Blood.

[6] Dale C.J., Thomson S., De Rose R., Ranasinghe C., Medveczky C.J., Pamungkas J., Boyle D.B., Ramshaw I.A., Kent S.J. (2006). Prime-boost strategies in DNA vaccines. Methods Mol. Med..

[7] Villarreal D.O., Talbott K.T., Choo D.K., Shedlock D.J., Weiner D.B. (2013). Synthetic DNA vaccine strategies against persistent viral infections. Expert Rev. Vaccines.

[8] Hung C.-F., Tsai Y.-C., He L., Wu T.-C. (2007). DNA vaccines encoding Ii-PADRE generates potent PADRE-specific CD4+ T-cell immune responses and enhances vaccine potency. Mol. Ther..

[9] Grgacic E.V.L., Anderson D.A. (2006). Virus-like particles: passport to immune recognition. Methods.

[10] Stoute J.A., Slaoui M., Heppner D.G., Momin P., Kester K.E., Desmons P., Wellde B.T., Garçon N., Krzych U., Marchand M. (1997). A preliminary evaluation of a recombinant circumsporozoite protein vaccine against *Plasmodium falciparum* malaria. RTS,S Malaria Vaccine Evaluation Group. N. Engl. J. Med..

[11] Douglas A.D., Williams A.R., Illingworth J.J., Kamuyu G., Biswas S., Goodman A.L., Wyllie D.H., Crosnier C., Miura K., Wright G.J. (2011). The blood-stage malaria antigen PfRH5 is susceptible to vaccine-inducible cross-strain neutralizing antibody. Nat. Commun..

[12] Farris E., Brown D.M., Ramer-Tait A.E., Pannier A.K. (2016). Micro- and nanoparticulates for DNA vaccine delivery. Exp. Biol. Med. (Maywood).

[13] Sambrook J., Fritsch E.F., Maniatis T. (1989). Molecular Cloning: A Laboratory Manual.

[14] Knudsen K.B., Northeved H., Kumar P.E., Permin A., Gjetting T., Andresen T.L., Larsen S., Wegener K.M., Lykkesfeldt J., Jantzen K. (2015). In vivo toxicity of cationic micelles and liposomes. Nanomedicine.

[15] Kaslow D.C., Biernaux S. (2015). RTS,S: toward a first landmark on the Malaria Vaccine Technology Roadmap. Vaccine.

[16] Tajiri K., Shimizu Y. (2015). Unsolved problems and future perspectives of hepatitis B virus vaccination. World J. Gastroenterol..

[17] de Oliveira C.I., Wunderlich G., Levitus G., Soares I.S., Rodrigues M.M., Tsuji M., del Portillo H.A. (1999). Antigenic properties of the merozoite surface protein 1 gene of *Plasmodium vivax*. Vaccine.

[18] Amorim K.N.S., Rampazo E.V., Antonialli R., Yamamoto M.M., Rodrigues M.M., Soares I.S., Boscardin S.B. (2016). The presence of T cell epitopes is important for induction of antibody responses against antigens directed to DEC205(+) dendritic cells. Sci. Rep..

[19] Chen L., Lopaticki S., Riglar D.T., Dekiwadia C., Uboldi A.D., Tham W.-H., O’Neill M.T., Richard D., Baum J., Ralph S.A., Cowman A.F. (2011). An EGF-like protein forms a complex with PfRh5 and is required for invasion of human erythrocytes by *Plasmodium falciparum*. PLoS Pathog..

[20] Agnandji S.T., Lell B., Soulanoudjingar S.S., Fernandes J.F., Abossolo B.P., Conzelmann C., Methogo B.G., Doucka Y., Flamen A., Mordmüller B., RTS,S Clinical Trials Partnership (2011). First results of phase 3 trial of RTS,S/AS01 malaria vaccine in African children. N. Engl. J. Med..

[21] Birkett A.J. (2016). Status of vaccine research and development of vaccines for malaria. Vaccine.

[22] Mordmüller B., Surat G., Lagler H., Chakravarty S., Ishizuka A.S., Lalremruata A., Gmeiner M., Campo J.J., Esen M., Ruben A.J. (2017). Sterile protection against human malaria by chemoattenuated PfSPZ vaccine. Nature.

[23] Reddy K.S., Amlabu E., Pandey A.K., Mitra P., Chauhan V.S., Gaur D. (2015). Multiprotein complex between the GPI-anchored CyRPA with PfRH5 and PfRipr is crucial for *Plasmodium falciparum* erythrocyte invasion. Proc. Natl. Acad. Sci. USA.

[24] Fotoran W.L., Santangelo R.M., Medeiros M.M., Colhone M., Ciancaglini P., Barboza R., Marinho C.R., Stábeli R.G., Wunderlich G. (2015). Liposomes loaded with P. falciparum merozoite-derived proteins are highly immunogenic and produce invasion-inhibiting and anti-toxin antibodies. J. Control. Release.

[25] Qiu Y., Guo L., Zhang S., Xu B., Gao Y., Hu Y., Hou J., Bai B., Shen H., Mao P. (2016). DNA-based vaccination against hepatitis B virus using dissolving microneedle arrays adjuvanted by cationic liposomes and CpG ODN. Drug Deliv..

[26] DeMuth P.C., Min Y., Huang B., Kramer J.A., Miller A.D., Barouch D.H., Hammond P.T., Irvine D.J. (2013). Polymer multilayer tattooing for enhanced DNA vaccination. Nat. Mater..

[27] Du Z., Munye M.M., Tagalakis A.D., Manunta M.D.I., Hart S.L. (2014). The role of the helper lipid on the DNA transfection efficiency of lipopolyplex formulations. Sci. Rep..

[28] Zavec A.B., Ota A., Zupancic T., Komel R., Ulrih N.P., Liovic M. (2014). Archaeosomes can efficiently deliver different types of cargo into epithelial cells grown in vitro. J. Biotechnol..

[29] Delpeyroux F., Peillon N., Blondel B., Crainic R., Streeck R.E. (1988). Presentation and immunogenicity of the hepatitis B surface antigen and a poliovirus neutralization antigen on mixed empty envelope particles. J. Virol..

[30] Wunderlich G., del Portillo H.A. (2000). Biochemical and immunological properties of a viral hybrid particle expressing the *Plasmodium vivax* merozoite surface protein 1 C-terminal region. Mol. Med..

[31] Mor G., Klinman D.M., Shapiro S., Hagiwara E., Sedegah M., Norman J.A., Hoffman S.L., Steinberg A.D. (1995). Complexity of the cytokine and antibody response elicited by immunizing mice with *Plasmodium yoelii* circumsporozoite protein plasmid DNA. J. Immunol..

[32] Becker S.I., Wang R., Hedstrom R.C., Aguiar J.C., Jones T.R., Hoffman S.L., Gardner M.J. (1998). Protection of mice against *Plasmodium yoelii* sporozoite challenge with *P. yoelii* merozoite surface protein 1 DNA vaccines. Infect. Immun..

[33] Wang L., Menting J.G., Black C.G., Stowers A., Kaslow D.C., Hoffman S.L., Coppel R.L. (2000). Differences in epitope recognition, isotype and titer of antisera to *Plasmodium falciparum* merozoite surface protein 4 raised by different modes of DNA or protein immunization. Vaccine.

[34] Moffat J.M., Cheong W.-S., Villadangos J.A., Mintern J.D., Netter H.J. (2013). Hepatitis B virus-like particles access major histocompatibility class I and II antigen presentation pathways in primary dendritic cells. Vaccine.

[35] Ita K. (2016). Transdermal delivery of vaccines—recent progress and critical issues. Biomed. Pharmacother..

[36] Rose A.H., Hoffmann F.W., Hara J.H., Urschitz J., Moisyadi S., Hoffmann P.R., Bertino P. (2015). Adjuvants may reduce in vivo transfection levels for DNA vaccination in mice leading to reduced antigen-specific CD8+ T cell responses. Hum. Vaccin. Immunother..

[37] Siegel F., Lu M., Roggendorf M. (2001). Coadministration of gamma interferon with DNA vaccine expressing woodchuck hepatitis virus (WHV) core antigen enhances the specific immune response and protects against WHV infection. J. Virol..

[38] DeMuth P.C., Li A.V., Abbink P., Liu J., Li H., Stanley K.A., Smith K.M., Lavine C.L., Seaman M.S., Kramer J.A. (2013). Vaccine delivery with microneedle skin patches in nonhuman primates. Nat. Biotechnol..

[39] Rosada R.S., de la Torre L.G., Frantz F.G., Trombone A.P., Zárate-Bladés C.R., Fonseca D.M., Souza P.R., Brandão I.T., Masson A.P., Soares E.G. (2008). Protection against tuberculosis by a single intranasal administration of DNA-hsp65 vaccine complexed with cationic liposomes. BMC Immunol..

[40] Hill A.V.S. (2011). Vaccines against malaria. Philos. Trans. R. Soc. Lond. B Biol. Sci..

[41] Tiwari S., Agrawal G.P., Vyas S.P. (2010). Molecular basis of the mucosal immune system: from fundamental concepts to advances in liposome-based vaccines. Nanomedicine (Lond.).

[42] Reddy K.S., Pandey A.K., Singh H., Sahar T., Emmanuel A., Chitnis C.E., Chauhan V.S., Gaur D. (2014). Bacterially expressed full-length recombinant *Plasmodium falciparum* RH5 protein binds erythrocytes and elicits potent strain-transcending parasite-neutralizing antibodies. Infect. Immun..

[43] Hjerrild K.A., Jin J., Wright K.E., Brown R.E., Marshall J.M., Labbé G.M., Silk S.E., Cherry C.J., Clemmensen S.B., Jørgensen T. (2016). Production of full-length soluble *Plasmodium falciparum* RH5 protein vaccine using a *Drosophila melanogaster* Schneider 2 stable cell line system. Sci. Rep..

[44] Boyle M.J., Reiling L., Feng G., Langer C., Osier F.H., Aspeling-Jones H., Cheng Y.S., Stubbs J., Tetteh K.K., Conway D.J. (2015). Human antibodies fix complement to inhibit *Plasmodium falciparum* invasion of erythrocytes and are associated with protection against malaria. Immunity.

[45] Wunderlich G., Moura I.C., del Portillo H.A. (2000). Genetic immunization of BALB/c mice with a plasmid bearing the gene coding for a hybrid merozoite surface protein 1-hepatitis B virus surface protein fusion protects mice against lethal *Plasmodium chabaudi chabaudi* PC1 infection. Infect. Immun..

[46] Trager W., Jensen J.B. (1976). Human malaria parasites in continuous culture. Science.

[47] Lelièvre J., Berry A., Benoit-Vical F. (2005). An alternative method for *Plasmodium* culture synchronization. Exp. Parasitol..

[48] Lambros C., Vanderberg J.P. (1979). Synchronization of *Plasmodium falciparum* erythrocytic stages in culture. J. Parasitol..

[49] Wilson D.W., Crabb B.S., Beeson J.G. (2010). Development of fluorescent *Plasmodium falciparum* for in vitro growth inhibition assays. Malar. J..

[50] Tonkin C.J., van Dooren G.G., Spurck T.P., Struck N.S., Good R.T., Handman E., Cowman A.F., McFadden G.I. (2004). Localization of organellar proteins in *Plasmodium falciparum* using a novel set of transfection vectors and a new immunofluorescence fixation method. Mol. Biochem. Parasitol..

[51] Moon J.J., Suh H., Li A.V., Ockenhouse C.F., Yadava A., Irvine D.J. (2012). Enhancing humoral responses to a malaria antigen with nanoparticle vaccines that expand Tfh cells and promote germinal center induction. Proc. Natl. Acad. Sci. USA.

