# Supplementary material for: DNA-Loaded Cationic Liposomes Efficiently Function as a Vaccine against Malarial Proteins
Source: Mol Ther Methods Clin Dev. 2017 Aug 23;7:1–10. doi: 10.1016/j.omtm.2017.08.004 (PMC5581859; doi:10.1016/j.omtm.2017.08.004)
Supplement: Document S1. Figures S1–S5 [file mmc1.pdf]

OMTM, Volume *7*

Supplemental Information

DNA-Loaded Cationic Liposomes Efficiently Function as a Vaccine against Malarial Proteins

Wesley L. Fotoran, Rachele Santangelo, Beatriz N.M. de Miranda, Darrell J. Irvine, and Gerhard Wunderlich

Supplementary Figure 1

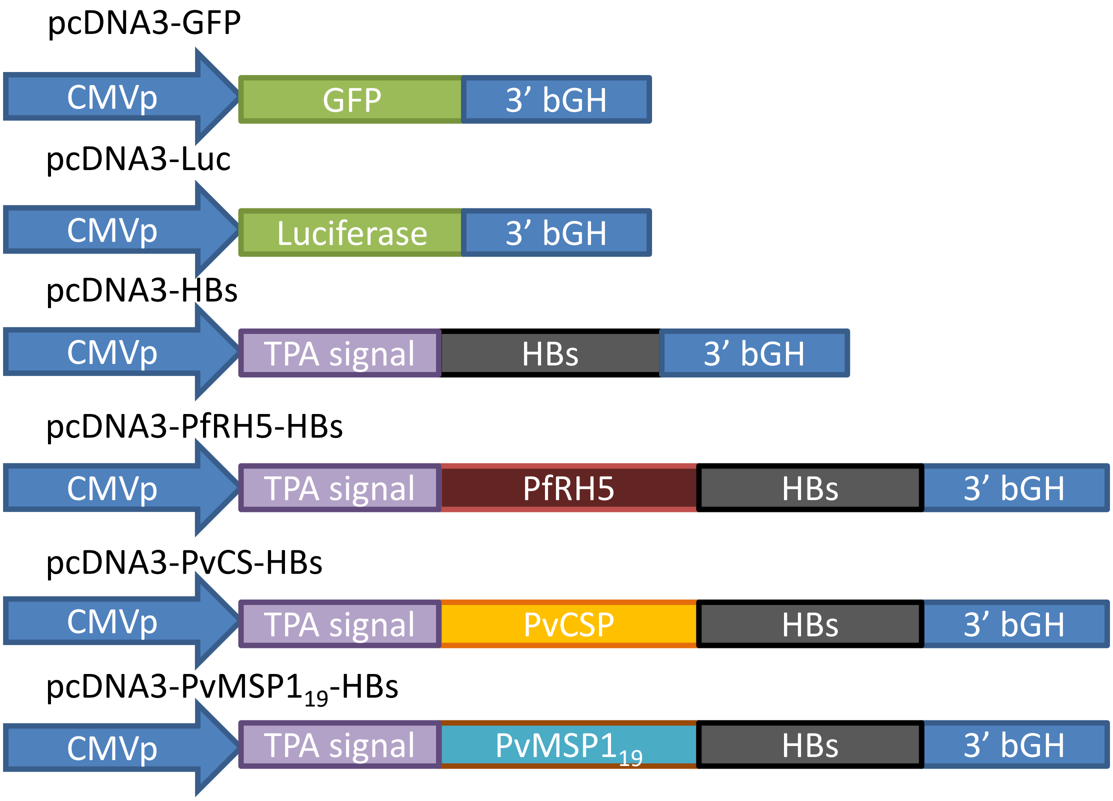

Supplementary Figure 1: Plasmids used for DNA entrapment in cationic liposomes. All plasmids were based on the pcDNA3 (Invitrogen) backbone. Plasmids using a TPA signal were constructed as described[13]. The codon-optimized gene for PfRH5 was a gift from Dr. Simon Draper and was cloned in frame with TPA and the HBs gene using BamH1 and EcoR1 sites at its termini. CMVp is the immediate-early Cytomegalovirus promoter, bGH is the bovine growth hormone terminator, and TPA signal is the 5`end of the tissue plasminogen activator secretion signal (encodes the first 26 amino acids of TPA)

Supplementary Figure 2

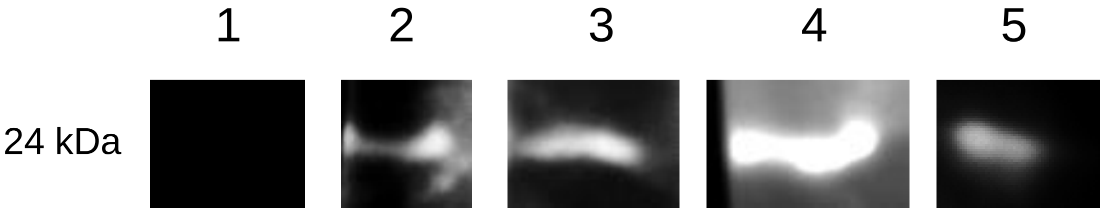

Supplementary Figure 2: Immunization of mice with different plasmids encoding the HBs domain induce antibodies against HBsAg. Plasmids were encapsulated in liposomes and administered i.p. in Balb/c mice. Then, pools of sera were used in 1:200 dilutions. Sera from mice immunized with the following plasmids were used: Lane 1: pcDNA3-GFP, 2: pcDNA3-HBs, 3: pcDNA3-PvCS-HBs, 4: pcDNA3-PvMSP1$_{19}$-HBs, 5: pcDNA3-PfRH5-HBs.

Supplementary Figure 3

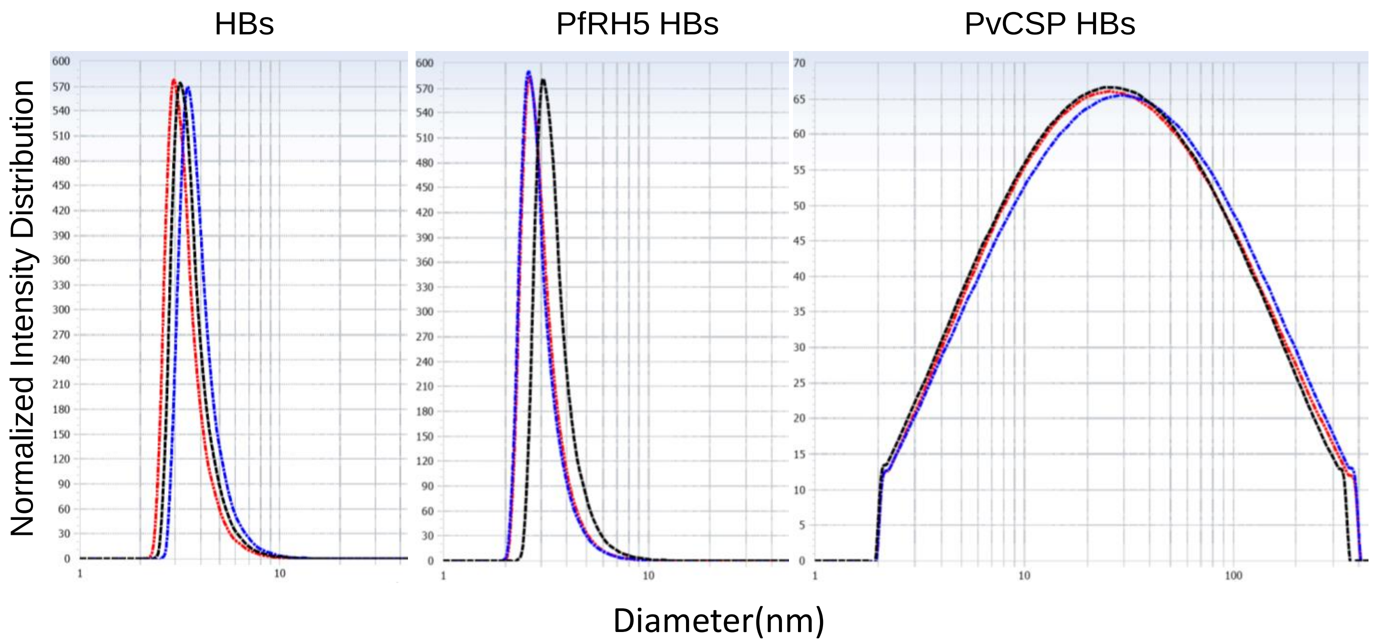

**Particle**	**Average size (nm)**	**PDI**
HBs	48	0.416
PfRH5-HBs	27.2	0.4
PvCSP-HBs	30.9	0.439

Size of particles found in the supernatant of CHO cells, transfected with pcDNA3-HBs, pcDNA3-PfRH5-HBs or pcDNA3-PvCSP-HBs. Culture supernatants were retrieved from transiently transfected cells as described and submitted to ultracentrifugation over a preformed sucrose gradient (20-60%). The fraction containing HBs (approximately 25-30% sucrose) was analyzed by Dynamic light scattering as described and the readouts and the observed particles sizes are shown. Curves in red, blue and black show outcomes for three independent samples of the same supernatant. PDI is Polydispersity Index.

A

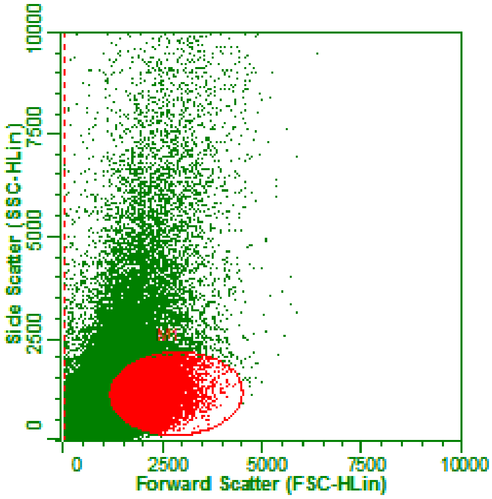

B

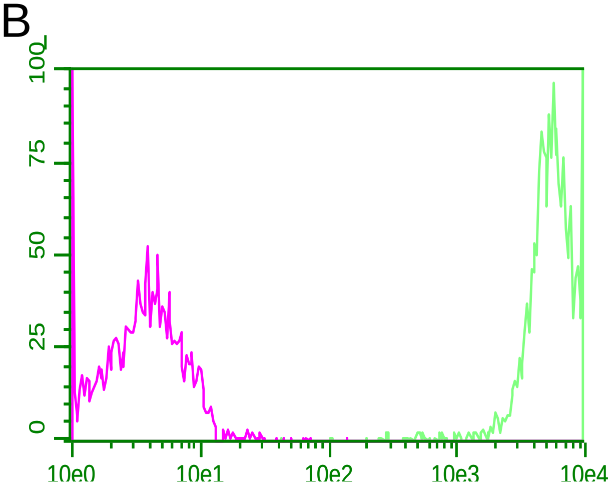

CFSE staining

In A, cytometer settings showing the gating of cells used either for CFSE staining or antibody labelling (see Supplementary Figure 5) is marked in red. The gate was set in order to avoid celular debris/dead cells (on the left margin outside the gate) and macrophages (above the gate) which possess another granulosity. The gate focuses on the lymphocyte profile in side/forward scatter analysis. In B, cells were then analysed for fluorescence in unstained form (magenta) or in CFSE-stained form (without stimulation). 50000 events per animal were acquired. The same gating was also applied for antibody labeling of cells (see Suppl. Figure 5).

Supplementary Figure 5:

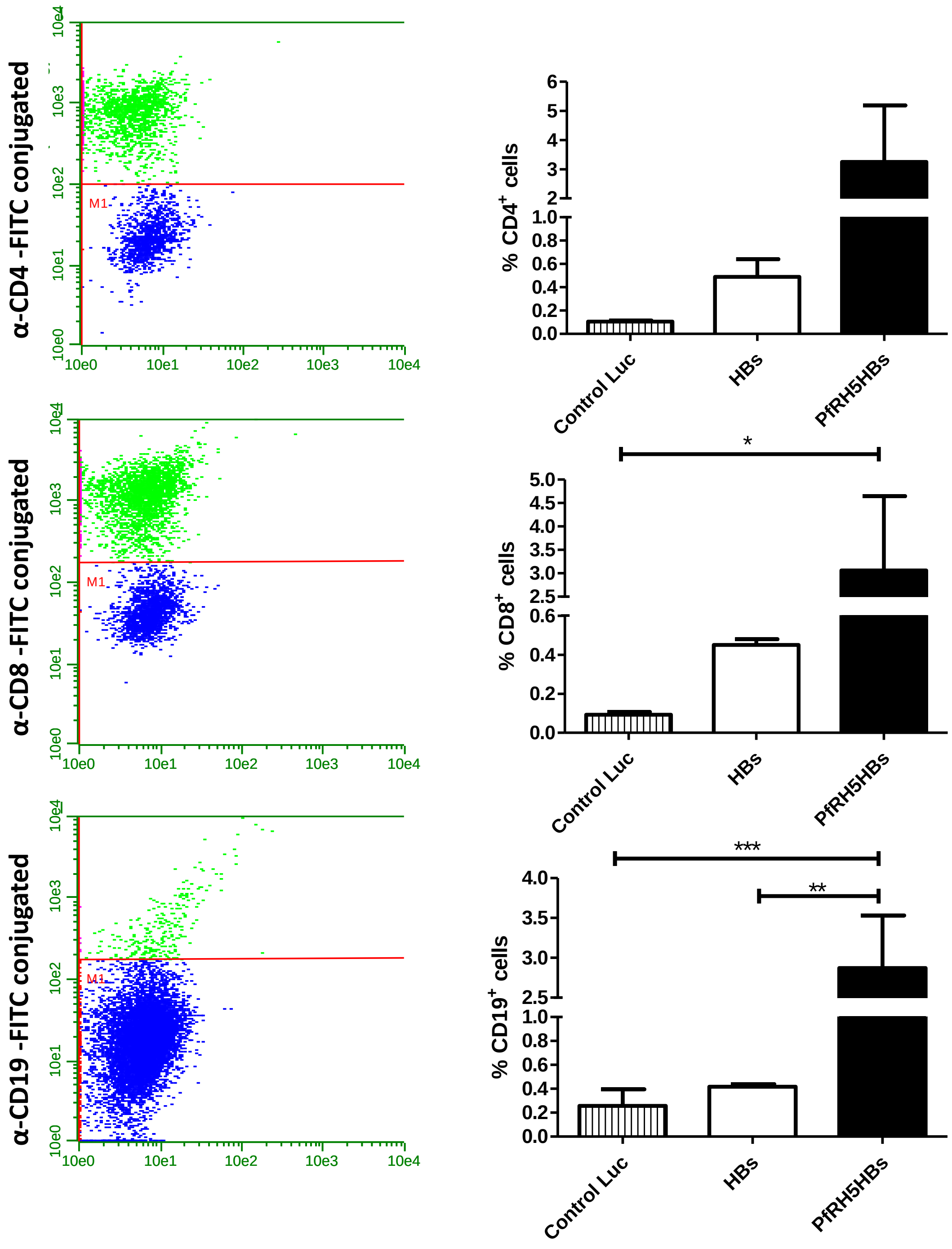

Expansion of subsets of lymphocytes upon stimulation of spleen cells from mice (n=5) immunized with liposomes loaded with pcDNA3-PfRH5-HBs and stimulated for 72 h with either pcDNA3-luc, pcDNA3-HBs or pcDNA3-PfRH5-HBs (all of which encapsulated in liposomes). The original gating and readouts are shown on the left (example of pre-challenge splenocytes from one mouse) and the evaluation is on the right. Significant differences are shown (ANOVA, *, $p<0.05$, **, $p<0.01$, ***, $p<0.005$). Note that splenocytes die under these conditions (high percentage of $CD4^+$/$CD8^+$/$CD19^+$ cells on the left, in green), but stimulated cells survive in different proportions (plots on the right). Supplementary Figure 4 shows the applied gating which avoided debris with fluorescence.
